# Higher Grade Glioma Increases the Risk of Postoperative Delirium: Deficient Brain Compensation Might Be a Potential Mechanism of Postoperative Delirium

**DOI:** 10.3389/fnagi.2022.822984

**Published:** 2022-04-13

**Authors:** Hua-Wei Huang, Xiao-Kang Zhang, Hao-Yi Li, Yong-Gang Wang, Bin Jing, You Chen, Mayur B. Patel, E. Wesley Ely, Ya-Ou Liu, Jian-Xin Zhou, Song Lin, Guo-Bin Zhang

**Affiliations:** ^1^Department of Critical Care Medicine, Beijing Tiantan Hospital, Capital Medical University, Beijing, China; ^2^Department of Neurosurgery, Beijing Tiantan Hospital, Capital Medical University, Beijing, China; ^3^Beijing Key Laboratory of Brain Tumor, Center of Brain Tumor, China National Clinical Research Center for Neurological Diseases (NCRC-ND), Beijing, China; ^4^School of Biomedical Engineering, Capital Medical University, Beijing, China; ^5^Department of Biomedical Informatics, Vanderbilt University, Nashville, TN, United States; ^6^Department of Electrical Engineering & Computer Science, Vanderbilt University, Nashville, TN, United States; ^7^Section of Surgical Sciences, Department of Surgery and Neurosurgery, Division of Trauma, Surgical Critical Care, and Emergency General Surgery, Vanderbilt University Medical Center, Nashville, TN, United States; ^8^Department of Hearing & Speech Sciences, Vanderbilt University Medical Center, Nashville, TN, United States; ^9^Geriatric Research, Education, and Clinical Center Service, Veterans Affairs Tennessee Valley Healthcare System, Nashville, TN, United States; ^10^Critical Illness, Brain Dysfunction, and Survivorship Center, Vanderbilt University Medical Center, Nashville, TN, United States; ^11^Department of Medicine, Division of Allergy, Pulmonary, and Critical Care Medicine, Vanderbilt University Medical Center, Nashville, TN, United States; ^12^Department of Radiology, Beijing Tiantan Hospital, Capital Medical University, Beijing, China

**Keywords:** postoperative delirium, brain cognitive compensation, frontal lobe glioma, structural and functional MRI, conceptual model

## Abstract

**Objective:**

The brain compensation mechanism in postoperative delirium (POD) has not been reported. We uncovered the mechanism by exploring the association between POD and glioma grades, and the relationship between preoperative brain structural and functional compensation with POD in patients with frontal glioma.

**Methods:**

A total of 335 adult patients with glioma were included. The multivariable analysis examined the association between tumor grade and POD. Then, 20 patients with left frontal lobe glioma who had presurgical structural and functional MRI data and Montreal Cognitive Assessment (MoCA) in this cohort were analyzed. We measured the gray matter volume (GMV) and functional connectivity (FC) in patients with (*n* = 8) and without (*n* = 12) POD and healthy controls (HCs, *n* = 29) to detect the correlation between the structural and functional alteration and POD.

**Results:**

The incidence of POD was 37.3%. Multivariable regression revealed that high-grade glioma had approximately six times the odds of POD. Neuroimaging data showed that compared with HC, the patients with left frontal lobe glioma showed significantly increased GMV of the right dorsal lateral prefrontal cortex (DLPFC) in the non-POD group and decreased GMV of right DLPFC in the POD group, and the POD group exhibited significantly decreased FC of right DLPFC, and the non-POD group showed the increasing tendency. Partial correlation analysis showed that GMV in contralesional DLPFC were positively correlated with preoperative neurocognition, and the GMV and FC in contralesional DLPFC were negatively correlated with POD.

**Conclusions:**

Our findings suggested that insufficient compensation for injured brain regions involving cognition might be more vulnerable to suffering from POD.

## Introduction

Postoperative delirium (POD) is a common surgical complication, with an incidence ranging from 11 to 51% (Rudolph and Marcantonio, 2011; Inouye et al., 2014). Although the delirium episodes are usually transient, it is associated with permanent adverse sequelae, including worsen long-term cognition and increased mortality (Rudolph and Marcantonio, 2011; Inouye et al., 2014; Huang et al., 2021). So far, the neural mechanism of POD remains obscure. Recent neuroimaging studies found that some brain structure or ultrastructure damage might be the neural substrates of vulnerability to POD (Cavallari et al., 2016, 2017; Racine et al., 2017). Therefore, in a multicentre observational study, van den Boogaard et al. (2012) found that the admission category of neurosurgery was an independent risk factor for the development of delirium. Several studies reported that the incidence of POD following neurosurgical procedures varied between 4 and 44% (Chen et al., 2014; Matano et al., 2017; Budėnas et al., 2018; Flanigan et al., 2018; Schubert et al., 2018; Tanaka et al., 2018; Wang et al., 2018; He et al., 2019; Morshed et al., 2019; Zipser et al., 2019). Our previous work has observed a unique phenomenon that in neurosurgical patients, the risk of POD was increased by approximately three-fold in those with malignant brain tumors (Wang et al., 2020). It seems that patients with faster lesion momentum are associated with greater POD risk. Probably because greater brain reorganization may occur when neuronal damage happens at a slower rate, while faster brain injury may leave little time for compensation and result in poor neurocognitive function (NCF) reserve (Noll et al., 2015; Klein, 2016; Wefel et al., 2016). We speculated that the growth kinetics and aggressiveness of brain tumors may regulate the development of POD through impacting brain compensation. Glioma comprises the vast majority of primary malignant brain tumors (Schwartzbaum et al., 2006). The glioma with different tumor grades would produce varying degrees of brain reorganization (Yuan B. et al., 2020). Therefore, glioma could be viewed as a model, which “knockout” the brain region involving certain function and also induce corresponding compensation. To preliminarily investigate the possible phenomenon of compensation mechanism that has not yet been reported in the occurrence of POD, we firstly performed a nested clinical data analysis of our previous prospective cohort study (Wang et al., 2020) to characterize the relationship between POD and tumor grades in patients with glioma. In addition, several important hub nodes involving the development of POD, such as the posterior cingulate cortex (PCC) and the prefrontal dorsolateral cortex (DLPFC), are located in the frontal lobe (Oh et al., 2019). Therefore, we then used multimodal MRI techniques to further verify the association between the brain’s structural or functional compensation and POD through the model of frontal lobe glioma. Here, frontal lobe glioma could be viewed as a model which “knocked out” the brain region associated development of POD and also induce corresponding compensation of cognitive function. We hypothesized that in patients with frontal lobe glioma, the structural and functional compensation induced by frontal lobe lesion might, to some extent, help the vulnerable brain resist perioperative stress and prevent POD. This is an indication that insufficient brain compensation might be the important pathological base of POD.

## Materials and Methods

This study was carried out in accordance with the latest version of the Declaration of Helsinki and the study procedures were approved by the institutional review board of Beijing Tiantan Hospital, Capital Medical University, Beijing, China (KY 2017-018-02). Written informed consent was obtained from all patients in this study.

### Study Design and Population

This was a nested analysis of a prospective cohort study of adult patients after elective intracranial surgery enrolled in the neurosurgical intensive care unit (ICU) (ClinicalTrials.gov NCT 03087838) of a university-affiliated, tertiary hospital in Beijing, China between March 2017 and February 2018 (Wang et al., 2020).

This study enrolled patients aged 18 and older, scheduled for elective intracranial surgery and with a centrally reviewed diagnosis of malignant glioma. The exclusion criteria were non-Chinese speakers, transsphenoidal surgery, cerebrospinal fluid (CSF) shunt and drainage surgery, pre-operative coma, pre-operative cognitive dysfunction due to PD or dementia, history of psychosis, could not be assessed for delirium, or unlikely to survive 24 h. Detailed information on perioperative clinical care is described in the Supplementary Material.

### Outcomes

The primary outcome was the occurrence of POD. The POD was assessed by two trained investigators twice a day using the confusion Assessment of the Method for the ICU (CAM-ICU) (Ely et al., 2001; Wang et al., 2013) during the first 72 h after ICU admission. First, the sedation status was determined by the Richmond Agitation-Sedation Scale (RASS), where a RASS score of at least −3 is necessary before the CAM-ICU can be continued to perform. CAM-ICU is positive when there is an “an acute onset of change or fluctuating mental status” and “inattention”, plus either “disorganized thinking” or “altered level of consciousness”. The presence of POD was defined as any positive CAM-ICU assessment.

### Exposure Variable – Tumor Grade

The primary exposure variable studied was a tumor grade. Specialist neuropathologists in our hospital made the histopathological diagnoses. Specifically, glioma graded for grades I to IV based on morphology, malignant behavior, and molecular parameters as defined in the 2016 WHO classification of tumors of the central nervous system (Louis et al., 2016). Grades I or II gliomas are defined as low-grade glioma and Grade III or IV are defined as high-grade (Louis et al., 2016). These data were collected retrospectively from the electronic medical record.

### Covariate Data

To adjust for confounding variables, potentially associated with the exposure variables of study outcomes, the following factors were included as covariates in our study: preoperative and intraoperative characteristics, such as patient demography, comorbidity, medical history, American Society of Anesthesiologists (ASA) physical status, anesthetic technique, use of midazolam as premedication, intraoperative use of opioids, intraoperative use of dexmedetomidine, intraoperative transfusion, episode of hypotension, and duration of operation were chosen from evidence-based and consensus-based risk factors for POD (Aldecoa et al., 2017). As very few neurosurgery-specific risk factors of POD in neurosurgical patients were reported, we collected the following brain tumor-specific factors, for example, tumor location, the limbic system involved, cortex involved, bihemispheric involved, largest tumor dimension, and extent of resection.

### Brain Imaging and Image Processing

#### Image Acquisition

Preoperative neuroimaging data obtained from subjects who performed preoperative high-resolution T1-weighed MRI and rest-state functional MRI (fMRI) (rs-fMRI) in the same type of MRI scanner (Philips Ingenia Elition, Amsterdam, Netherlands) were analyzed. Previous studies have reported that the frontal lobe might be one of the important brain regions involved in POD (Oh et al., 2019), therefore, we chose the patients with glioma in the frontal lobe as the subject of our further research. However, in our studies, there were very few patients with glioma in the right frontal lobe who completed comprehensive required imaging examinations. To avoid the effect of tumor location, only the patients with glioma in the left frontal lobe were included and analyzed. For all subjects, T1-weighted images were acquired with the following parameters: axial magnetization prepared rapid gradient echo (MPRAGE) sequence; repetition time (TR) = 1,900 msec; echo time (TE) = 3 msec; flip angle (FA) = 8°, inversion time (TI) = 900 msec; field of view (FOV) = 256 × 256 mm; matrix size = 256 × 192; voxel size = 1 × 1 × 1 mm^3^; and scanning time = 7 min 47 s. The rs-fMRI images were acquired using the following parameters: TR = 2,000 msec; TE = 30 msec; FA = 90°, FOV = 192 × 192 mm; matrix size = 64 × 64; thickness = 3 mm; slice number = 33; and scanning time = 8 min. All subjects were instructed to relax, keep their eyes close, and not think about anything. The healthy control (HC) group consisted of 29 age-, sex-, and education-matched healthy volunteers who were examined in the same scanner as the patients.

#### Data Preprocessing

Structural image processing was conducted using Statistical Parametric Mapping 12 (SPM 12) and Computational Anatomy toolbox 12 (CAT 12) running in the MATLAB environment (MathWorks Inc., CA, United States). The procedure started at manual reorientation to the anterior commissure with SPM12. Then, using the CAT 12, 3D T1-weighted images were segmented into GM, white matter (WM), and CSF. Next, non-brain tissue was removed by an automated brain extraction procedure with segmentation. The extracted GM images were normalized to the Montreal Neurological Institute-152 (MNI-152) standard space with the voxel size as 1.5 mm and, then, smoothed with an 8-mm full width at half-maximum (FWHM) Gaussian Kernel. The generated smoothened GM images were subjected to the following statistical analyses.

The rs-fMRI data were preprocessed using SPM12 and the Data Processing & Analysis of Brain Imaging toolbox (DPABI). The preprocessing steps mainly included a standard pipeline, including removing the first 10 scans, slice-timing correction, realignment, unwarping, spatial normalization, regressing out nuisance covariates, filtering 0.01−0.1 Hz temporal bandpass, and scrubbing. Then, regions showing significant differences in GM volumes between patient groups were selected as seeds to calculate functional connectivity (FC). Pearson’s correlation coefficients were computed between the mean time series of seeds for the rest of the whole brain (based on BN246 parcellation). To improve the normality, the correlation coefficients were transformed into Fisher’s z scores and the results were displayed as a connectivity matrix for each participant.

### Assessment of Preoperative Cognitive Function

The patients included in the neuroimaging study needed to assess the preoperative cognitive function by administration of the Montreal Cognitive Assessment (MoCA). The MoCA total score was obtained by summing up seven subscale scores, including visual-spatial abilities, name objects, attention, language, abstraction, delayed recall, and orientation. The MoCA is scored out of 30. Patients scoring below 26 were identified as having cognitive impairment.

### Statistical Analysis

All clinical data statistical analyses were performed using SAS version 9.3 (SAS Institute, United States). The Kolmogorov-Smirnov test was used to test the normality of all variables. Continuous data were presented as mean ± SD or median and interquartile range; binary data were summarized *via* frequency and percentage. Comparisons of continuous data were made using a two-tailed independent *t*-test or a Mann-Whitney *U* test, and categorical data were compared by a Pearson chi-square or Fisher’s exact test, as appropriate. The baseline patient characteristics, intraoperative characteristics, and brain tumor-specific factors that were significant in the univariable analysis at a threshold of *P* < 0.05, along with strong confounders of age, sex, and education, were entered into a backward multivariable logistic regression model. Before any multivariable analysis, collinearity among covariates was assessed using the variance inflation factor; variables with a variance inflation factor > 3 were excluded. We then used a multivariable to characterize the risk-adjusted association between the primary exposure of glioma tumor grade and the primary outcome of POD. Odds ratios (ORs) and their 95% CIs were used to assess the independent contributions of significant factors. A *P*-value of less than 0.05 was considered statistically significant. The Hosmer-Lemeshow test was used to determine the appropriateness of the model.

Our data were obtained from our previous well-design, prospective cohort study, and all of the data were collected according to the protocol, therefore, there was no missing data in the present nested investigation study (Wang et al., 2020). The sample size for this nested cohort study was determined by the number of patients with available POD and tumor grade. However, we had also determined that a sample size of 52 patients would be necessary to detect a difference in POD incidence between patients with high-grade glioma and patients with low-grade glioma using a χ^2^ test with a type I error of 0.05 and a power of >80%, assuming a low-grade glioma prevalence of approximately 30%, a high-grade glioma prevalence of 70%, and a POD incidence of 10% in the patients with low-grade glioma and 48% in the high-grade patients.

The statistical analysis of the neuroimaging data was calculated in the SPM 12 and the DPABI software. A voxel-wise two-sample *t*-test was carried out to compare the GM volume (GMV) differences between the patient groups (POD and non-POD groups) in SPM 12. Considering the small sample size, we performed a non-parametric permutation test with age, sex, and total intracranial volumes (TIVs) as covariates and used a threshold-free cluster enhancement family-wise error (TFCE-FWE) corrected two-tailed *P* < 0.05 at cluster level and cluster size *k* > 40. We used classification of the Cohen’s *d* effect size as suggested by Cohen (Bowring et al., 2021), indicating 0.5 as a “medium” effect, 0.8 as “large,” and 1.2 as “very large.” To explore the differences of seed-based FC matrices between the POD and non-POD groups, two-sample *t*-tests were analyzed by using DPABI and GRETNA software running in the MATLAB environment (MathWorks Inc., CA, United States). Age, sex, and head motion were included as covariates to control the possible influences of these factors on the results. For fMRI data, the significant threshold was set as a less conservative uncorrected thresholds *P* < 0.01 due to the small sample size. The mean GMV and seed-based FC in brain regions or networks that significantly changed in patient groups were extracted from the HC group and a two-sample *t*-test was performed with the patient groups to further investigate whether these changes were compensation or not. Partial correlations were applied to confirm the relationship between the brain structural and (or) functional compensation and preoperative cognitive function as well as POD. The significance level was set at a two-sided *P* < 0.05.

## Results

A total of 335 patients with glioma were included in this analysis. The POD was diagnosed in 125 patients (37.3%), of whom 78 (62.4%), 27 (21.6%), and 20 (16%) were classified as hypoactive, hyperactive, and mixed subtypes, respectively.

### Impact of Tumor Grade – Univariable and Multivariable Analysis

**FIGURE 1:**
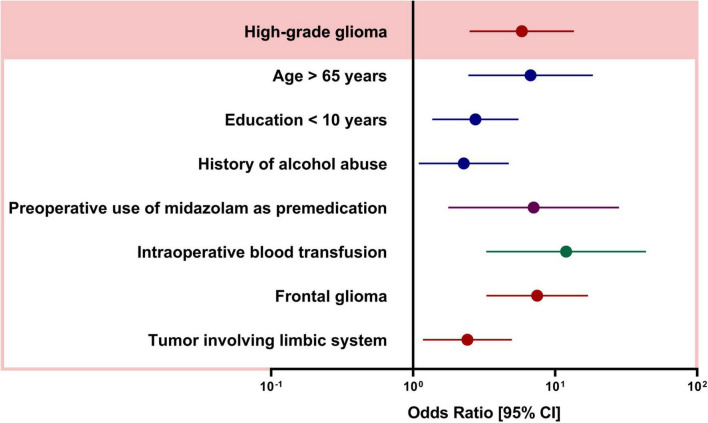
Adjusted odds ratios (OR) from multivariable logistic regression. OR and [95% CIs] are calculated from the model described in Methods.

The univariable comparison of baseline patient characteristics, intraoperative characteristics, and brain tumor-specific factors between the patients with and without POD is shown in Table 1. Compared to patients without POD, POD was more likely to occur in patients with age > 65 years (*P* < 0.001), lower education level (*P* < 0.001), ASA III-IV (*P* < 0.001), history of alcohol abuse (*P* = 0.04), and history of hypertension and stroke (both *P* < 0.001). In terms of intraoperative characteristics, patients with POD more frequently used midazolam as premedication (*P* < 0.001), used fentanyl only for anesthesia maintenance (*P* = 0.008) and had more often intraoperative transfusion (*P* < 0.001). In terms of tumor characteristics, patients with POD were more likely to have higher grade glioma (*P* < 0.001), tumors located in the frontal (*P* < 0.001), and occipital lobe (*P* = 0.029), tumors involving the limbic system (*P* < 0.001), bihemispheric tumor (*P* < 0.001), and larger tumor (*P* = 0.013). We observed no collinearity among covariates (variables inflation factors below 3) and thus included all these variables into a multivariable model. Figure 1 shows the adjusted ORs for POD from the final statistical model. Patients with high-grade glioma had approximately six times the odds (OR 5.826, CI 2.501–13.570, *P* = 0.000) of experiencing POD. In addition, subjects with age > 65 years (OR 6.720, CI 2.450–18.433, *P* < 0.001), education level < 10 years (OR 2.746, CI 1.364–5.528, *P* = 0.005), and history with alcohol abuse (OR 2.274, CI 1.097–4.714, *P* = 0.027) was more likely to develop POD. Preoperative use of midazolam as premedication (OR 7.064, CI 1.772–28.157, *P* = 0.006) and intraoperative blood transfusion (OR 11.957, CI 3.273–43.673, *P* < 0.001) remained strongly associated with POD. In the neurosurgery-specific factors, patients with frontal glioma had a seven-fold increase in the odds of POD (OR 7.486, CI 3.280–17.087, *P* < 0.001), and those with a tumor involving the limbic system also had an increased odd of POD (OR 2.415, CI 1.175–4.964, *P* = 0.016). The results of the Hosmer-Lemeshow test showed that the model fitted the data well (*P* = 0.431).

**TABLE 1:** Univariable logistic regression of preoperative patients’ factors, intraoperative patients characteristics and tumor characteristics.

Variable	Entire population (*n* = 335)	Delirium (*n* = 125)	No delirium (*n* = 210)	*P*-value†	OR (95% CI)†
**Preoperative patient factors**					
**Patient characteristics**					
Age > 65 years (n, %)	53(15.8%)	38(30.4%)	15(7.1%)	<0.001	5.678 (2.968–10.865)
Male (n, %)	177(52.8%)	65(52.0%)	112(53.3%)	0.813	
Education < 10 years (n, %)	164(49%)	34(27.2%)	130(61.9%)	<0.001	1.694 (1.418–2.024)
ASA physical status				<0.001	11.128 (2.888–35.520)
I – II (n, %)	316(94.3%)	109(87.2%)	207(98.6%)		
III – IV (n, %)	19(5.7%)	16(12.8%)	3(1.4%)		
History of alcohol abuse (n, %)	81(24.2%)	41(32.8%)	40(19.0%)	0.004	2.074 (1.248–3.448)
History of smoking (n, %)	104(31.0%)	45(36%)	59(28.1%)	0.130	
**Relevant co-morbid condition**					
Hypertension (n, %)	64(19.1%)	41(32.8%)	23(11.0%)	<0.001	3.968 (2.240–7.030)
Coronary artery disease (n, %)	6(1.8%)	0(0%)	6(2.9%)	0.139	
Diabetes (n, %)	31(9.3%)	16(12.8%)	15(7.1%)	0.084	
Stroke (n, %)	9(2.7%)	9(7.2%)	0(0%)	<0.001	1.078 (1.026–1.132)
History of antiepileptics (n, %)	60(17.9%)	23(18.4%)	37(17.6%)	0.857	
**Intraoperative characteristics**					
Use of midazolam for premedication (n, %)	26(7.8%)	21(16.8%)	5(2.4%)	<0.001	8.279 (3.035–22.582)
Anesthesia technique				0.151	
TIVA (n, %)	92(27.5%)	40(32.0%)	52(24.8%)		
Balanced anesthesia (n, %)	243 (72.5)	85(68.0%)	158(75.2%)		
Use of fentanyl only for anesthesia maintain (n, %)	22(6.6%)	14(11.2%)	8(3.8%)	0.008	3.185 (1.296–7.825)
Intraoperative use of dexmedetomidine (n, %)	43(12.8%)	18(14.4%)	25(11.9%)	0.509	
Intraoperative transfusion (n, %)	29(8.7%)	25(20.0%)	4(1.9%)	<0.001	12.875 (4.363–37.995)
Anaesthesia duration > 4 h (n, %)	244(72.8%)	89(71.2%)	155(73.8%)	0.604	0.877 (0.535–1.438)
**Tumor characteristics**					
WHO grading (n, %)				<0.001	1.744 (1.516–2.007)
I – II	98(29.3%)	10(8.0%)	88(41.9%)		
III –IV	237(70.7%)	115(92.0%)	122(58.1%)		
**Location (n, %)**					
Frontal lobe	120(35.8%)	67(53.6%)	53(25.2%)	<0.001	1.653 (1.331–2.054)
Temporal lobe	58(17.3%)	20(16.0%)	38(18.1%)	0.624	
Parietal lobe	11(3.3%)	3(2.4%)	8(3.8%)	0.484	
Occipital lobe	9(2.7%)	0(0%)	9(4.3%)	0.029	
Insular lobe	20(6.0%)	11(8.8%)	9(4.3%)	0.092	
Other (e.g., Cerebellum, brainstem)	117(34.9%)	24(19.2%)	93(44.3%)	<0.001	
Limbic system involved (n, %)	100(29.9%)	57(45.6%)	43(20.5%)	<0.001	3.255 (2.002–5.293)
Bihemispheric tumor (n, %)	22(6.6%)	16(12.8%)	6(2.9%)	<0.001	4.991 (1.898–13.121)
Largest tumor dimension > 6 cm (n, %)	82(24.5%)	40(32.0%)	42(20.0%)	0.013	1.882 (1.135–3.121)
Extent of resection (n, %)				0.436	
Gross-total resection	202(60.3%)	72(57.6%)	130(61.9%)		
Subtotal and partial resection	133(39.7%)	53(42.4%)	80(38.1%)		

*ASA, American Society of Anesthesiologists; CI, confidence interval; ICU, intensive care unit; OR, odds ratio; WHO, World Health Organization. †P-values are based on the Wald statistic from univariate logistic regression. ORs are shown when P-values are < 0.05.*

### Neuroimaging Data Analyses

#### Demographic Characteristics

As shown in Table 2, the patients in POD group (*n* = 8) were older (50.25 ± 1.48 vs. 41.76 ± 9.22, *P* < 0.001) and had lower education level (8.25 ± 4.92 vs. 12.24 ± 1.35, *P* = 0.070) than HCs (*n* = 29), while there was no significant difference in age (44.27 ± 11.17 vs. 41.76 ± 9.22, *P* = 0.526) and education (12 ± 4.90 vs. 12.24 ± 1.35, *P* = 0.995) between non-POD group and HC subjects. Compared to the HC subjects, neither the non-POD group nor the POD group showed significant differences in total intracranial volume (all *P* > 0.05). In addition, there was no significant difference in tumor volume between the non-POD group and the POD group (*P* > 0.05).

**TABLE 2:** Demographic and clinical characteristic among patient with left frontal lobe glioma and healthy controls.

Variable	Postoperative delirium (*n* = 8)	No postoperative delirium (*n* = 12)	HC (*n* = 29)
Age (years)	50.25 (1.48)	44.27 (11.17)	41.76 (9.22) ***
Sex (M/F)	4/4	5/7	15/14
Education level (years)	8.25 (4.92)	12 (4.90)	12.24 (1.35)
Handedness (R/L)	8/0	12/0	29/0
Total intracranial volume (cm^3^)	1378.54 (88.66)	1442.81 (119.86)	1441.35 (120.78)
Tumor volume (cm^3^)	69.34 (54.95)	45.40 (35.78)	NA

Data are presented as mean (standard deviation, SD). F, females; HC, healthy controls; L, left; R, right; M, males; NA, not applicable. ***represent P < 0.001 when POD vs. HC.

#### Comparison of Gray Matter Volume Between the Postoperative Delirium, Non-postoperative Delirium Patients, and the Healthy Controls

**FIGURE 2:**
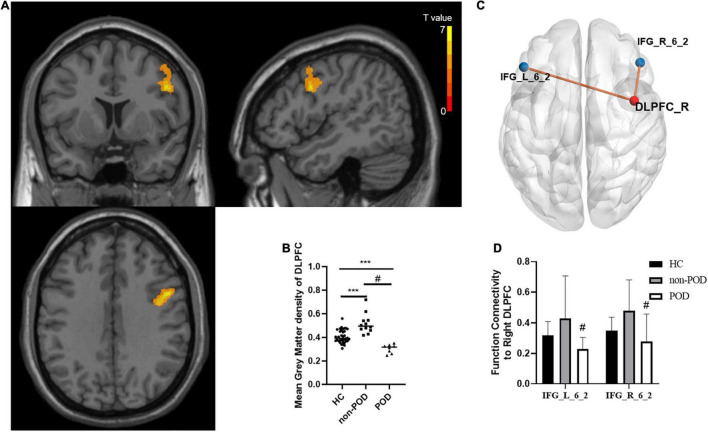
Comparisons of gray matter (GM) volume as well as functional connectivity between healthy controls (HCs) and patients with left frontal lobe glioma with and without delirium. **(A)** Spatial distribution of the GM volume abnormalities between delirium group and no delirium group. The yellow area presents brain region with significantly increased GM density calculated by voxel-based morphology (VBM) analysis (Voxel size = 1.5 mm^3^, cluster size = 114, Permutation test + TFCE, *P* < 0.001, Cohen’s *d* = 2.95). The color bar shows the *T* value. These samples show enhanced GM volume in the dorsal lateral prefrontal cortex (DLPFC)_R. **(B)** The comparison of mean GM density in the right DLPFC between HCs, postoperative delirium (POD) group, and non-POD group. Bars show the mean; error bars show the SE. **(C)** The differences of functional connectivity based on DLPFC_R between the non-POD group and POD group are shown in MNI-152 space with a *P*-value threshold of 0.01. **(D)** The comparison of *z* value of functional connectivity between DLPFC_R and IFG_L _6_2, as well as DLPFC_R and IFG_R _6_2 between HC group, POD group, and non-POD group. *** compared with HC, Mann-Whitney *U* test, *P* < 0.01, # compared with non-POD, Mann-Whitney *U* test, *P* < 0.01. DLPFC, dorsolateral prefrontal cortex; GM, gray matter; HC, health control; POD, postoperative delirium; R, right; VBM, voxel-based morphometry.

As shown in Figure 2A, compared with the POD group, the non-POD group displayed significantly increased GM volume in brain regions BA6 which is part of DLPFC (*P* < 0.05, TFCE-FWE corrected and cluster size = 114 voxels, Cohen’s *d* = 2.95). No significant difference in GM volume was found in other regions. As shown in Figure 2B, compared with the HC group, the patients showed the significantly increased GM volume of the right DLPFC in the non-POD group (*P* < 0.001, Cohen’s *d* = 1.42) and decreased GM volume of the right DLPFC in the POD group (*P* < 0.001, Cohen’s *d* = −1.95).

#### Comparison of Seed-Based Functional Connectivity Between the Postoperative Delirium, Non-postoperative Delirium Patients, and the Healthy Controls

Using the different brain regions between the POD and non-POD groups obtained from voxel-based morphometry (VBM) analysis as seeds, we found that the functional connectivity to the right DLPFC increased in the non-POD group compared with the POD group, and the connective module was shown in Figure 2C. Compared to the POD group, the functional connectivity increased from the right DLPFC to the bilateral inferior frontal sulcus (IFG_L/R_6_2) in the non-POD group (*P* < 0.01, Cohen’s *d* = 0.91 and 1.04, respectively). Meanwhile, compared with HCs, the POD group exhibited significantly decreased functional connectivity, and the non-POD showed functional connectivity with increasing tendency but they did not achieve statistical significance (Figure 2D).

### Correlation Between Structural and Functional Indices and Postoperative Delirium

**FIGURE 3:**
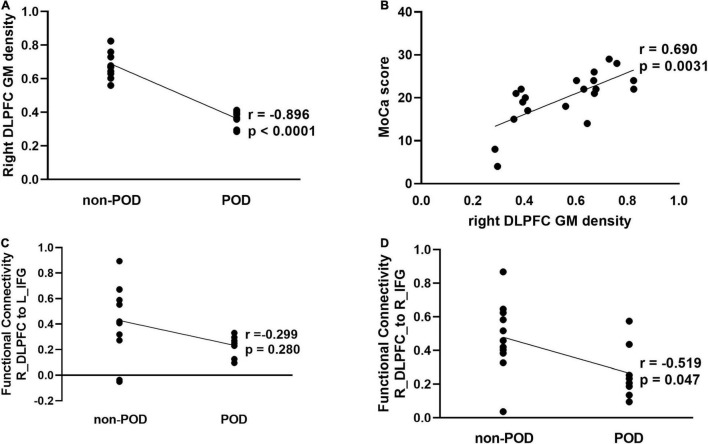
Correlation analysis between structural and functional alterations in the contralesional DLPFC and POD in patients with left frontal lobe glioma. **(A)** Partial correlation analysis between mean GM density of right DLPFC and POD (*r* = –0.896, *P* < 0.001). **(B)** Partial correlation analysis between mean GM density of right DLPFC and preoperative montreal cognitive assessment (MOCA) scores (*r* = 0.690, *P* = 0.003). **(C)** Partial correlation analysis between function connectivity from DLPFC_R to IFG_L_6_2 and POD (*r* = –0.299, *P* = 0.280). **(D)** Partial correlation analysis between function connectivity from DLPFC_R to IFG_R_6_2 and POD (*r* = –0.519, *P* = 0.047). DLPFC, dorsolateral prefrontal cortex; GM, gray matter; HC, health control; IFG, inferior frontal sulcus; L, left; POD, postoperative delirium; R, right.

The partial correlation analysis after controlling for the effects of age, sex, and education showed a significant correlation between the mean GMV in right DLPFC and POD (*r* = −0.896, *P* < 0.0001) (Figure 3A) and a significantly positive correlation between increased GMV in the right DLPFC and preoperative neurocognition measured by MOCA scores (Figure 3B). In addition, we also found that a significant correlation between the FC between the contralateral DLPFC and contralateral inferior frontal gyrus and POD (*r* = −0.519, *P* = 0.047), whereas no significant correlation in FCs between the contralateral DLPFC and ipsilateral inferior frontal gyrus (*r* = −0.299, *P* = 0.280) (Figures 3C,D).

## Discussion

In our study, we detected that higher grade glioma was independently and strongly associated with POD. Other significant predictors of POD were older age, lower education, alcohol abuse, use of midazolam as premedication, blood transfusion, and glioma located in the frontal lobe and involving the limbic system. Our clinical data suggested that brain lesion momentum might affect the occurrence of POD. We validated primitively in small sample neuroimaging data to explore the underlying mechanism and found that increased GMV and FC in the contralateral DLPFC (even increased than healthy controls) was associated with decreased risk of POD in patients with left frontal glioma. Therefore, we speculated that insufficient brain compensation may be the important pathological base of POD from the model of frontal lobe glioma.

We have first reported the association between the risk of POD and the malignant degree of glioma. Our findings are similar to prior studies reporting NCF deficits in patients with glioma (Noll et al., 2015; Wefel et al., 2016). Noll et al. (2015) found preoperative NCF impairment to be more frequent even after controlling for lesion volume in patients with high-grade glioma in the left temporal lobe. Wefel et al. (2016) found that IDH1-wild type tumors which were more aggressive than IDH1-mutant also resulted in more frequent preoperative NCF impairment. Presumably, slower-growing glioma could allow greater time for migration of focal neurologic functions to nearby or contralateral structures, so patients may have milder NCF deficits (Klein, 2016). Otherwise, patients with fast-growing gliomas have no sufficient time for compensation and may have a serious NCF deficit. The recent neuroimaging studies strengthen the evidence linking cognitive compensation with brain reorganization (Hu et al., 2020; Liu D. et al., 2020; Yuan T. et al., 2020). Hu et al. (2020) showed that reorganization of contralateral GMV was associated with cognition in patients with unilateral temporal glioma. Liu Y. et al. (2020) suggested that contralesional homotopic functional connectivity in patients with unilateral temporal glioma was positively associated with cognitive functional compensation. Previous works demonstrated preoperative cognitive reserve is associated with the risk of POD (Feinkohl et al., 2017; Humeidan et al., 2021). Therefore, our finding supports the contention that insufficient brain compensation for injured brain regions involving cognition might be more vulnerable to experiencing POD.

**FIGURE 4:**
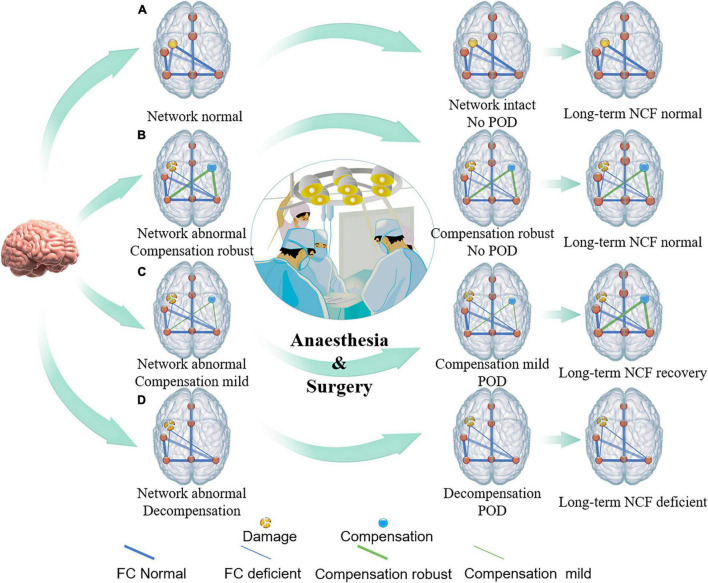
Conceptual model of relationship between brain damage, brain compensation, and POD. The conceptual model illustrates that premorbid integrity of individual brain regions (yellow intact or broken dots on the left side of brain) and/or brain connectivity (thick or thin blue line), as well as robustness of brain compensation (yellow dots and green line on the right side of brain), may relate to the POD incidence under conditions of heightened stress from surgery. **(A)** Individuals with intact brain structure and function, such as healthy young people, can accommodate surgical stress, and, thus, do not suffer from POD. **(B)** Individuals with impaired brain structure and (or) function but robust compensation, such as some chronic brain-injured patients, including brain benign tumor and patients with high education level et al may also be less likely to suffer POD. **(C)** Individuals with impaired brain structure and (or) function but insufficient compensation, such as relative fast brain-injured patients including brain tumors with low malignancy are prone to have the symptoms of delirium after suffering from a surgical strike. As the stressor resolves, brain compensation may continue to occur, and, thus, long-term cognitive function will be restored or might be less affected. **(D)** When brain structure and (or) function were impaired but compensation, or decompensation, has not yet been established, such as highly malignant brain tumor, acute stroke, traumatic brain injury, Alzheimer’s disease, etc., individuals with greater vulnerability experience greater POD incidence, duration, and severity.

The neural mechanisms of POD mostly focused on brain structural and functional injury, but rarely on brain compensation (Cavallari et al., 2016, 2017; Shioiri et al., 2016). Several studies suggested that cognitive compensatory mechanisms might involve delaying progression or relieving symptoms of Alzheimer’s disease and depression (Bai et al., 2019; Rajji, 2019). Therefore, we proposed an Impaired Neural Plasticity Theory hypothesis of POD (Figure 4). Specifically, POD is the consequence of brain network breakdown induced by perioperative stressors in individuals with pre-existing deficient brain connectivity and neuroplasticity. Impaired cognitive networks might be the neural pathological basis. However, reorganized networks to compensate for focal disruptions perhaps help the vulnerable brain resist acute insults. The phenomenon that patients with acute brain injury such as stroke, who did not have enough time to compensate, are at high risk of POD might bring us closer to explaining our theory hypothesis (Duceppe et al., 2019; Shaw et al., 2019). Then, we used a small set of neuroimaging data of patients with left frontal glioma to primarily test the association between POD and brain compensation, and found that increased structure and function in the contralateral DLPFC was negatively associated with risk for POD. Glioma invading the unilateral frontal lobe could induce contralesional brain reorganization within the posterior cognitive control network (CCN) (Liu Y. et al., 2020). The DLPFC is one of the important nodes within CCN (Breukelaar et al., 2020), therefore, in our study, increased structure and function in the contralateral DLPFC might be brain compensation induced by frontal lobe glioma. Given that a major function of the DLPFC is associated with execution (Kumar et al., 2017), our results suggested that though the ipsilateral executive control area was disrupted by tumor, the compensation of the contralateral executive control area might help the vulnerable brain become robust to resist stresses and prevent POD.

In addition, we identified another two tumor-specific risk factors – frontal lobe glioma and glioma involving the limbic system. Previous fMRI studies showed an abnormal interaction between the default mode network (DMN) and central executive network (CEN) during an episode of delirium (Oh et al., 2019). The posterior cingulate cortex and DLPFC are the hub nodes of DMN and CEN, respectively, and both of them are located in the frontal lobe (Oh et al., 2019). Therefore, patients with frontal lobe glioma have seven times increased risk of developing POD. The limbic system, such as corpus callosum and hippocampus, could be linked to impairment in integrative, visuospatial, and memory function, which often characterizes the delirium syndrome (Cavallari et al., 2016; Shioiri et al., 2016). The hippocampus is one of the most important brain regions involved in the stress-response system (Kurumaji et al., 2011). Therefore, its damage could make the brain more vulnerable to resist stress. The corpus callosum played an important role in the inter-hemispheric compensation of sensorimotor functioning in the case of unilateral brain damage (Wang et al., 2012). Therefore, lesions involving the limbic system might make corpus callosum connecting fibers mediated compensation become unstable.

In accordance with previous studies, we also found that the use of benzodiazepines is a risk factor of POD (Aldecoa et al., 2017). Several studies suggested that γ-aminobutyric acid-ergic inhibition is related to functional plasticity and behavioral improvement (Jacob, 2019). A randomized controlled trial in patients with eloquent area glioma, who had no major neurologic deficits at baseline, found that midazolam might augment or reveal motor dysfunction through inhibiting brain compensatory networks (Lin et al., 2016). Therefore, disrupting cognitive compensatory networks may be one of the potential mechanisms of POD caused by benzodiazepines.

Some study limitations warrant mention. First, because of limited neuroimaging data, we only analyzed the data of 20 patients with glioma in the left frontal lobe and did not investigate the phenomenon in patients with glioma in the right frontal lobe and more other brain regions. Therefore, whether the contralesional neuroanatomical and function changes are compensation induced by brain tumors or the neural substrates of vulnerability to POD, do not allow conclusions to be drawn. Secondly, our restricted enrolment of patients with glioma may not be generalizable to the entire surgical population. However, POD might be the result of a failed “stress test” for the brain, revealing underlying neurological injury in the presence of a surgical strike (Vlisides and Mashour, 2019). Brain injury and brain compensation always go together. Therefore, insufficient brain compensation may be the universal pathological base of POD.

In summary, we found that higher grade glioma was independently associated with risk of POD and hypothesize that insufficient brain compensation may be the important pathological base of POD. Furthermore, we used multimodal MRI to primarily validate this hypothesis in patients with frontal lobe glioma. Our work should enrich the pathogenesis of POD and provide a more theoretical basis for clinical interventions of POD.

## Data Availability Statement

The raw data supporting the conclusions of this article will be made available by the authors, without undue reservation.

## Ethics Statement

The studies involving human participants were reviewed and approved by the Institutional Review Board of Beijing Tiantan Hospital, Capital Medical University. The patients/participants provided their written informed consent to participate in this study.

## Author Contributions

H-WH: conceptualization, funding acquisition, project administration, and writing – original draft. X-KZ: project administration, data curation, and visualization. H-YL: project administration, formal analysis, and visualization. Y-GW: resources and investigation. BJ: software. YC, MP, and EE: writing – review and editing. Y-OL: resources. J-XZ: supervision. SL: resources and supervision. G-BZ: conceptualization, funding acquisition, supervision, and methodology. All authors contributed to the article and approved the submitted version.

## Conflict of Interest

The authors declare that the research was conducted in the absence of any commercial or financial relationships that could be construed as a potential conflict of interest.

## Publisher’s Note

All claims expressed in this article are solely those of the authors and do not necessarily represent those of their affiliated organizations, or those of the publisher, the editors and the reviewers. Any product that may be evaluated in this article, or claim that may be made by its manufacturer, is not guaranteed or endorsed by the publisher.
