# Supplementary material for: Higher Grade Glioma Increases the Risk of Postoperative Delirium: Deficient Brain Compensation Might Be a Potential Mechanism of Postoperative Delirium
Source: Front Aging Neurosci. 2022 Apr 13;14:822984. doi: 10.3389/fnagi.2022.822984 (PMC9045131; doi:10.3389/fnagi.2022.822984)
Supplement: Supplementary file 1 [file Data_Sheet_1.pdf]

frontiers

Supplementary Material

1 Perioperative clinical care

1.1 Anesthesia and Surgery

Perioperative anesthetic management for subjects was at the discretion of the attending anesthesiologist. All operations were performed under general balanced anesthesia or total intravenous anesthesia (TIVA). Routinely, anesthetic agents included induction with midazolam; analgesia with sufentanil, remifentanil or (and) fentanyl; neuromuscular blockade with rocuronium or vecuronium. Anesthesia was induced with propofol and fentanyl, sufentanil or remifentanil and maintained with propofol and/or sevoflurane or isoflurane and/or dexmedetomidine. Subjects received standard intraoperative neurophysiological monitoring according to recent international recommendations(Gertsch et al. 2019). Intraoperative hypotension is defined as systolic blood pressure (SBP) below 95mmHg, or in the case of a baseline SBP below 119 mmHg, a decrease of SBP by more than 20% of baseline value(Wang et al. 2020). Brain tissue relaxation was assessed by neurosurgeons using a four-point scale reported previously(Rozet et al. 2007). Fluids, osmotherapy, blood products and vasoactive infusions were managed at the discretion of the attending anesthesiologist in communication with the neurosurgeons.

1.2 Clinical Management of ICU Admission

We adopted the selective ICU admission strategy in our institution, that was, only high-risk patients should receive ICU-specific monitoring and treatment(Hanak et al. 2014). Specifically, anesthesiologist and neurosurgeon discuss postoperative ICU admission using criteria including, but not limited to, an age over 65 years, an American Society of Anesthesiologists' physical status (ASA) of at least three, the presence of a large tumor, pre-operative consciousness impairments, an anticipated prolonged procedure and an anticipated delayed extubation, major intra-operative hemorrhage or brain swelling, or severe cardiorespiratory instability during the attempt of emergence. After ICU admission, subjects receive standard ICU cardiorespiratory monitoring. Blood sample is obtained to measure serum concentrations of sodium, potassium, creatinine and albumin, blood glucose and hematocrit. Neurologic examinations are performed every hour by trained nurses. Postoperative computed tomography (CT) scans are usually performed within 6 hours after operation. Most patients receive patient-controlled intravenous analgesia (PCIA). If necessary, use of fentanyl, midazolam or propofol for sedation and analgesia is at the discretion of the intensivists in our department. After monitoring overnight, the patients are usually transferred to a general care floor the next morning, contingent on clinical stability and the absence of need for any ICU-specific interventions.

References

Gertsch JH, Moreira JJ, Lee GR, Hastings JD, Ritzl E, Eccher MA, Cohen BA, Shils JL, McCaffrey MT, Balzer GK, et al. 2019. Practice guidelines for the supervising professional: intraoperative neurophysiological monitoring. J Clin Monit Comput. 33,175-183. Epub 2018/10/31.doi:10.1007/s10877-018-0201-9

Hanak BW, Walcott BP, Nahed BV, Muzikansky A, Mian MK, Kimberly WT, Curry WT. 2014. Postoperative intensive care unit requirements after elective craniotomy. World Neurosurg. 81,165-172. Epub 2012/11/28.doi:10.1016/j.wneu.2012.11.068

Rozet I, Tontisirin N, Muangman S, Vavilala MS, Souter MJ, Lee LA, Kincaid MS, Britz GW, Lam AM. 2007. Effect of equiosmolar solutions of mannitol versus hypertonic saline on intraoperative brain relaxation and electrolyte balance. Anesthesiology. 107,697-704. Epub 2007/12/13.doi:10.1097/01.anes.0000286980.92759.94

Wang CM, Huang HW, Wang YM, He X, Sun XM, Zhou YM, Zhang GB, Gu HQ, Zhou JX. 2020. Incidence and risk factors of postoperative delirium in patients admitted to the ICU after elective intracranial surgery: A prospective cohort study. Eur J Anaesthesiol. 37,14-24. Epub 2019/08/30.doi:10.1097/eja.0000000000001074
